# Astragaloside IV Reduces Sorafenib-Induced Cardiotoxicity by Inhibiting Apoptosis Through the STAT3/HIF-1α/Bcl-2 Signaling Pathway

**DOI:** 10.3390/ijms27104243

**Published:** 2026-05-10

**Authors:** Lei Wang, Baonian Liu, Qianhui You, Hao Cai, Tianyun Huang, Yangjunpeng Lei, Hao Wang, Yelan Yao, Shuijin Shao, Haidong Guo

**Affiliations:** Department of Anatomy, School of Integrative Medicine, Shanghai University of Traditional Chinese Medicine, Shanghai 201203, China; wongleeedu@163.com (L.W.); liubaonian@shutcm.edu.cn (B.L.); nk31ultraver@outlook.com (Q.Y.); mygame-ch@163.com (H.C.); grace_hty@126.com (T.H.); lyjp2002@163.com (Y.L.); wanghao199608@126.com (H.W.); yaoye_lan@163.com (Y.Y.)

**Keywords:** astragaloside IV, sorafenib-induced cardiotoxicity, STAT3/HIF-1α/Bcl-2 pathway, apoptosis

## Abstract

Sorafenib is a first-line tyrosine kinase inhibitor for malignant tumor treatment, yet its clinical application is greatly restricted by unavoidable cardiotoxicity. Astragaloside IV is a natural compound with prominent cardiovascular protective effects. We first carried out modeling studies including network pharmacology, human proteome microarray screening, molecular docking, and molecular dynamics simulation. Network pharmacology highlighted the hypoxia-inducible factor-1 signaling pathway as a key route; the integrated approach further identified signal transducer and activator of transcription 3 as a novel direct binding target of astragaloside IV with high binding stability. In a mouse model of chronic sorafenib-induced cardiotoxicity, astragaloside IV significantly improved cardiac function, and attenuated myocardial fibrosis, oxidative damage, and cardiomyocyte apoptosis. Mechanistically, astragaloside IV reduced the expression of signal transducer and activator of transcription 3 and hypoxia-inducible transcription factor-1α, and elevated the expression of B-cell lymphoma 2. In cellular experiments, astragaloside IV protected HL-1 cardiomyocytes against sorafenib-induced cytotoxicity and apoptosis through the same signaling pathway. This study confirms that astragaloside IV alleviates sorafenib-induced cardiotoxicity by inhibiting cardiomyocyte apoptosis via targeting the signal transducer and activator of transcription 3/hypoxia-inducible transcription factor-1α/B-cell lymphoma 2 pathway, providing a promising strategy for clinical prevention of chemotherapy-related cardiac injury.

## 1. Introduction

Sorafenib (SOR), a multi-targeted tyrosine kinase inhibitor (TKI), has become a first-line treatment for renal cell carcinoma [1,2], hepatocellular carcinoma [3,4], and differentiated thyroid carcinoma [5,6]. However, its clinical utility is severely limited by prominent cardiotoxicity [7], including hypertension, left ventricular dysfunction, myocardial ischemia, and even life-threatening events, including cardiac arrest in patients without pre-existing heart disease [8]. Despite its widespread use, the mechanisms underlying SOR-induced myocardial injury remain incompletely elucidated.

Given the significant cardiotoxicity of SOR and the limitations of current protective strategies, exploring safe and effective interventions is paramount [9]. Therefore, we are focusing our attention on the field of traditional Chinese medicine [10]. Astragalus membranaceus (Huang Qi) is a widely utilized herb known for its tonifying and protective properties [11]. Its representative bioactive component, astragaloside IV (3-O-beta-D-xylopyranosyl-6-O-beta-D-glucopyranosyl-cycloastragenol, AST), has demonstrated therapeutic potential [12]. In the cardiovascular field, AST has shown efficacy in mitigating myocardial apoptosis, fibrosis [13], ischemia–reperfusion injury, and hypertrophy [14]. To further explore the molecular mechanism of AST, we employed network pharmacology, an integrative approach for predicting drug targets and mechanisms; it is especially useful for studying the compounds of traditional medicine. Combined with HuProt^TM^ human proteome microarray analysis, our preliminary data suggested that the HIF-1 signaling pathway might be involved in AST’s action against SOR-induced cardiotoxicity.

Investigating the role of specific signaling pathways is crucial for understanding such complex pathological processes. The signal transducer and activator of transcription 3 (STAT3) is a key molecule in the hypoxia-inducible factor-1 (HIF-1) signaling network [15,16,17,18]. Notably, emerging evidence suggests that dysregulated STAT3 activation contributes to SOR-induced myocardial damage [19], making it a potential therapeutic target for cardioprotection [20,21,22,23,24,25,26]. In the context of drug-induced cardiac injury, STAT3 may trigger high expression of hypoxia-inducible transcription factor-1α (HIF-1α), another key factor in cardiovascular pathology. HIF-1α can further influence the expression of the downstream B-cell lymphoma-2 (Bcl-2) [27,28]. However, the specific function of the STAT3/HIF-1α/Bcl-2 pathway in SOR-induced cardiotoxicity remains unclear, and whether AST can modulate this interplay to exert cardioprotection requires further investigation.

In the present study, we first performed network pharmacology, molecular docking, and HuProt™ human proteome microarray to identify the direct molecular targets of AST against SOR-induced cardiotoxicity. Based on these predictive results, our core hypothesis is that AST alleviates SOR-induced cardiotoxicity by directly targeting STAT3 and subsequently modulating the HIF-1α/Bcl-2 signaling pathway, thereby inhibiting cardiomyocyte apoptosis. To test this hypothesis, we established a mouse model of chronic SOR-induced cardiotoxicity to evaluate the in vivo cardioprotective effects of AST and performed in vitro experiments in HL-1 cardiomyocytes to confirm the underlying molecular mechanism. This study aims to clarify the protective mechanism of AST against SOR-induced cardiotoxicity and provide a potential strategy for the clinical prevention and treatment of TKI-related cardiac injury.

## 2. Results

### 2.1. Analysis of the Mechanism of AST Against SOR-Induced Cardiotoxicity Using Network Pharmacology

**Figure 1:**
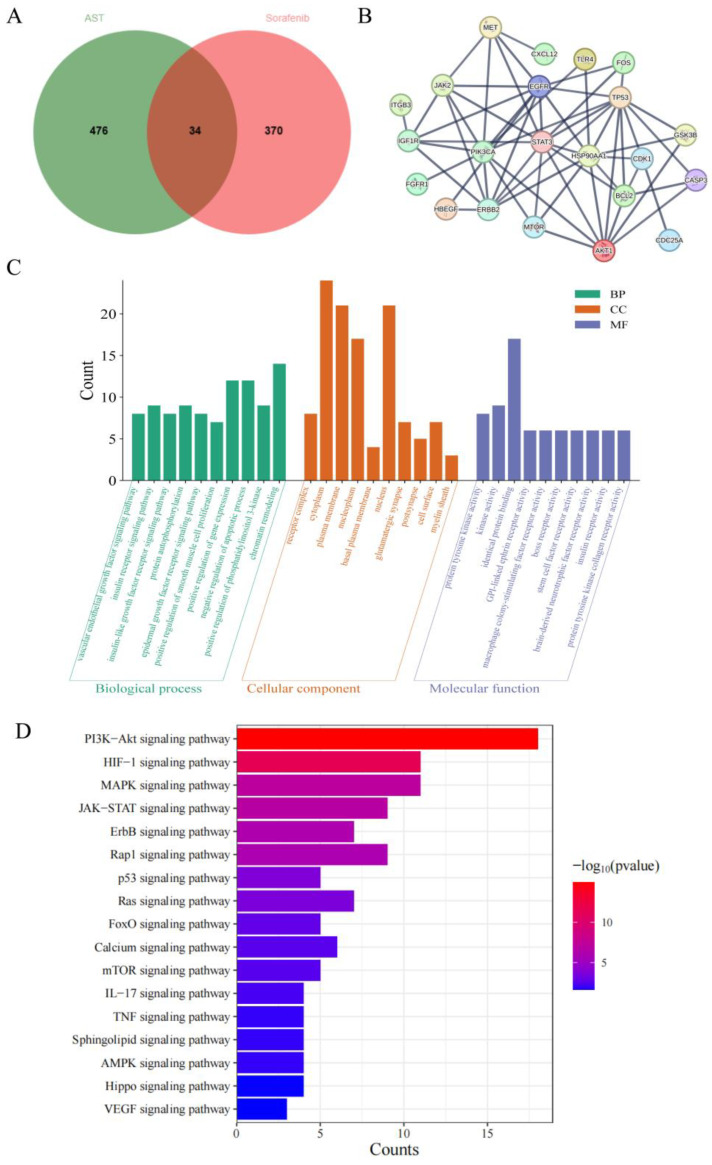
Network pharmacology analysis reveals potential targets and pathways of AST against SOR-induced injury. (**A**) The Venn diagram illustrating the overlapping targets of AST- and SOR-induced cardiac injury. (**B**) PPI network. (**C**) Top 10 enriched GO terms in BP, CC, and MF categories. (**D**) Top 17 significantly enriched KEGG pathways.

To identify the molecular targets and signaling pathways mediating the cardioprotective effects of AST, network pharmacology analysis was performed. Potential targets related to AST- and SOR-induced cardiac injury were retrieved from multiple databases. After removing duplicates and standardizing gene names, a total of 510 AST-related targets and 404 SOR injury-related targets were obtained. A Venn diagram identified 34 overlapping targets, which were defined as core targets for subsequent mechanistic exploration (Figure 1A). To prioritize key targets among these candidates, we further constructed a protein–protein interaction (PPI) network utilizing the STRING database (Figure 1B). Topological analysis, including degree, betweenness centrality, and closeness centrality, revealed several hub targets with high connectivity, such as STAT3, Bcl-2, and TP53. Subsequently, Gene Ontology (GO) enrichment analysis showed enrichment in biological processes such as “positive regulation of gene expression” and “negative regulation of apoptotic process” (Figure 1C). For cellular components, targets were primarily localized to the “cytoplasm” and “plasma membrane”. Key molecular functions included “identical protein binding” and “kinase activity”. Finally, Kyoto Encyclopedia of Genes and Genomes (KEGG) pathway enrichment analysis mapped the core targets to signaling pathways, identifying 17 significant entries (Figure 1D). The top-ranked pathways included PI3K-Akt, HIF-1, MAPK, and JAK-STAT signaling, which are closely associated with cardiomyocyte survival, oxidative stress response, and apoptosis.

### 2.2. Identification of STAT3 as a Direct AST-Binding Protein

**Figure 2:**
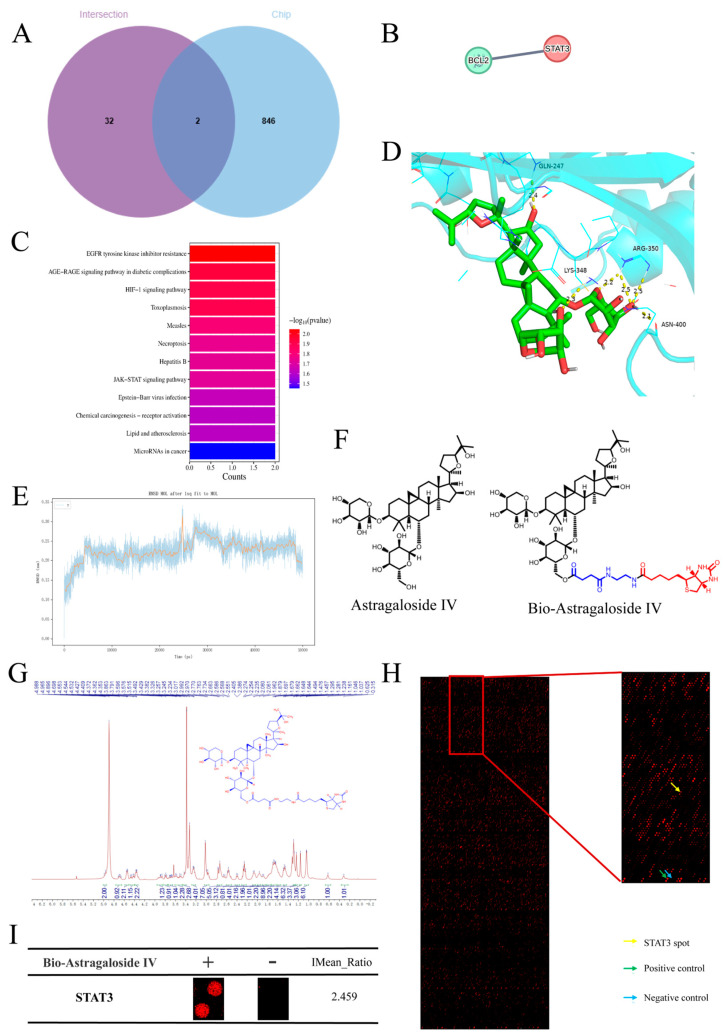
Validation of STAT3 as a direct binding target of AST. (**A**) Venn diagram of potential AST targets, SOR injury−related genes, and genes screened from the human proteome microarray. (**B**) Core targets. (**C**) KEGG pathway. (**D**) Molecular docking result between AST and STAT3. (**E**) RMSD of the STAT3 backbone during molecular dynamics simulation. (**F**) Chemical structures of AST and its bioactive form. (**G**) Mass spectrum of Bio−AST−STAT3 binding complex. (**H**) Representative images of the protein array showed a positive control (marked with a green arrow), a negative control (marked with a blue arrow), and a STAT3 spot (marked with a yellow arrow) in a selected region of the microarrays. (**I**) Enlarged images and IMean_Ratio value for STAT3.

To validate whether the hub target STAT3 is a direct binding partner of AST (rather than an indirect downstream regulator), we combined multi-omics screening and biophysical validation approaches. First, we integrated three datasets—AST-related targets, SOR injury-related genes, and hits from a human proteome microarray (probed with biotin-labeled AST)—and a Venn diagram (Figure 2A), which narrowed the candidate direct target to STAT3. A PPI sub-network (Figure 2B) further confirmed that STAT3 and Bcl-2 are core targeted proteins, a key anti-apoptotic protein identified in the earlier network analysis, reinforcing the link between STAT3 and AST’s anti-apoptotic effects. Through KEGG comprehensive analysis (Figure 1D, Figure 2C), it was concluded that the HIF-1 signaling pathway may be the crucial axis for AST to prevent and treat SOR-induced cardiotoxicity.

Next, molecular docking simulations were performed to predict the binding mode between AST and STAT3 (Figure 2D). The results demonstrated that AST binds stably to STAT3 with a binding energy of −8.3 kcal/mol, generating hydrogen bonds with residues GLN-247, ARG-350, LYS-348, and ASN-400. To confirm the stability of this binding complex, we conducted a 100 ns molecular dynamics simulation (Figure 2E): the root mean square deviation (RMSD) of the STAT3 backbone remained below 0.2 nm throughout the simulation, indicating that the AST-STAT3 complex maintained a stable conformation.

Experimental validation was then conducted using a human proteome microarray (Figure 2F–I): the array was incubated with biotin-labeled AST (Bio-AST), and signal detection revealed a specific, high-intensity binding signal for STAT3 (IMean_Ratio = 2.459, significantly higher than background). Finally, mass spectrometry of the immunoprecipitated Bio-AST–protein complex confirmed the presence of both AST and STAT3 peptides, providing direct experimental evidence that STAT3 is a direct binding target of AST.

### 2.3. AST Attenuates SOR-Induced Cardiotoxicity in Mice

**Figure 3:**
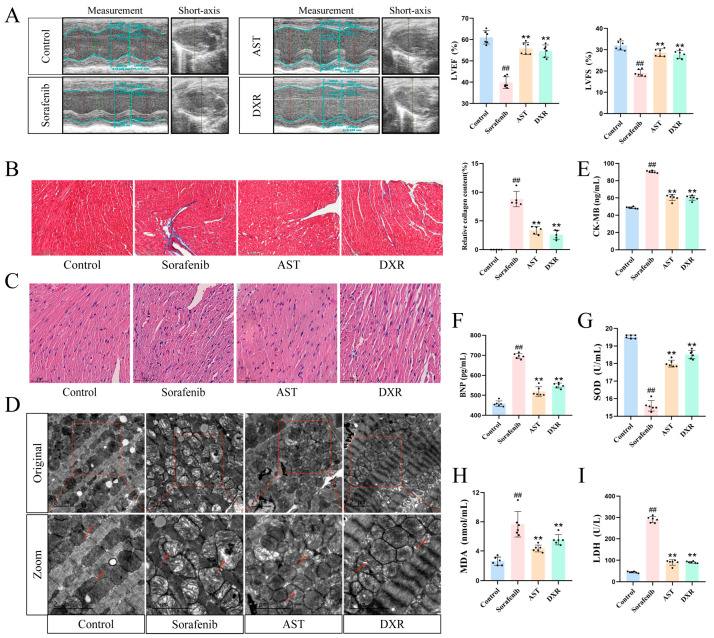
Astragaloside IV attenuates sorafenib-induced cardiotoxicity in mice. (**A**) Representative M-mode echocardiograms (parasternal short-axis view) and quantitative analysis of LVEF and LVFS, *n* = 6. (**B**) Masson’s representative trichrome-stained cardiac sections (scale bar = 200 μm), *n* = 5. (**C**) Representative HE-stained cardiac sections (scale bar = 50 μm), *n* = 6. (**D**) Representative TEM images (red arrows: damaged mitochondria; scale bar = 200 nm), *n* = 6. (**E**–**I**) Quantitative analysis of relative collagen content, CK-MB, BNP, SOD, MDA, and LDH levels. ## *p* < 0.01 vs. control; ** *p* < 0.01 vs. SOR.

Compared with the control group, SOR treatment significantly reduced left ventricular ejection fraction (LVEF) (control: 61.06 ± 3.14% vs. sorafenib: 39.83 ± 2.72%; ## *p* < 0.01) and left ventricular fractional shortening (LVFS) (control: 31.99 ± 2.14% vs. sorafenib: 19.09 ± 1.57%; ## *p* < 0.01). Treatment with AST (AST LVEF: 55.82 ± 2.82%; AST LVFS: 28.62 ± 1.79%; ** *p* < 0.01 vs. sorafenib) or positive control drug dexrazoxane (DXR) significantly restored cardiac systolic function (DXR LVEF: 54.62 ± 2.87%; DXR LVFS: 27.81 ± 1.85%; ** *p* < 0.01 vs. sorafenib) (Figure 3A). Histopathological examination showed that SOR induced marked myocardial disarray, cytoplasmic vacuolization, and interstitial fibrosis (control: 0 ± 0% vs. sorafenib: 8.82 ± 1.35%; ## *p* < 0.01), which were notably ameliorated by the administration of AST (AST: 3.36 ± 0.68%; DXR: 2.61 ± 0.78%; ** *p* < 0.01 vs. sorafenib) (Figure 3B,C). Transmission electron microscopy revealed that SOR caused severe mitochondrial cristae rupture and vacuolation, while AST pretreatment maintained mitochondrial structural integrity (Figure 3D). Biochemical analysis demonstrated that SOR significantly increased serum levels of CK-MB (control: 48.69 ± 1.02 ng/mL vs. sorafenib: 90.3 ± 1.13 ng/mL; ## *p* < 0.01), BNP (control: 458.05 ± 13.66 pg/mL vs. sorafenib: 696.12 ± 13.12 pg/mL; ## *p* < 0.01), MDA (control: 2.63 ± 0.56 nmol/mL vs. sorafenib: 7.66 ± 1.74 nmol/mL; ## *p* < 0.01), and LDH (control: 44.64 ± 3.38 U/L vs. sorafenib: 290.18 ± 14.32 U/L; ## *p* < 0.01) and decreased SOD activity (control: 19.53 ± 0.09 U/mL vs. sorafenib: 15.59 ± 0.3 U/mL; ## *p* < 0.01). These changes were significantly reversed by AST treatment (AST CK-MB: 60.32 ± 3.4 ng/mL; AST BNP: 518.7 ± 23.77 pg/mL; AST MDA: 4.34 ± 0.47 nmol/mL; AST LDH: 89.59 ± 12.8 U/L; AST SOD: 18 ± 0.2 U/mL; ** *p* < 0.01 vs. sorafenib) (Figure 3E–I).

### 2.4. Astragaloside IV Ameliorates Sorafenib-Induced Myocardial Injury Through STAT3/HIF-1α/Bcl-2 Signaling Pathway-Mediated Inhibition of Apoptosis

To further clarify the molecular mechanism of AST against SOR-induced cardiotoxicity, we designed additional animal groups including a STAT3 agonist ML115 treatment group and an AST combined with ML115 group on the basis of the control, SOR, and AST groups in the previous in vivo experiment. This grouping scheme aims to verify whether AST alleviates myocardial injury by inhibiting cardiomyocyte apoptosis through the STAT3/HIF-1α/Bcl-2 signaling pathway.

**Figure 4:**
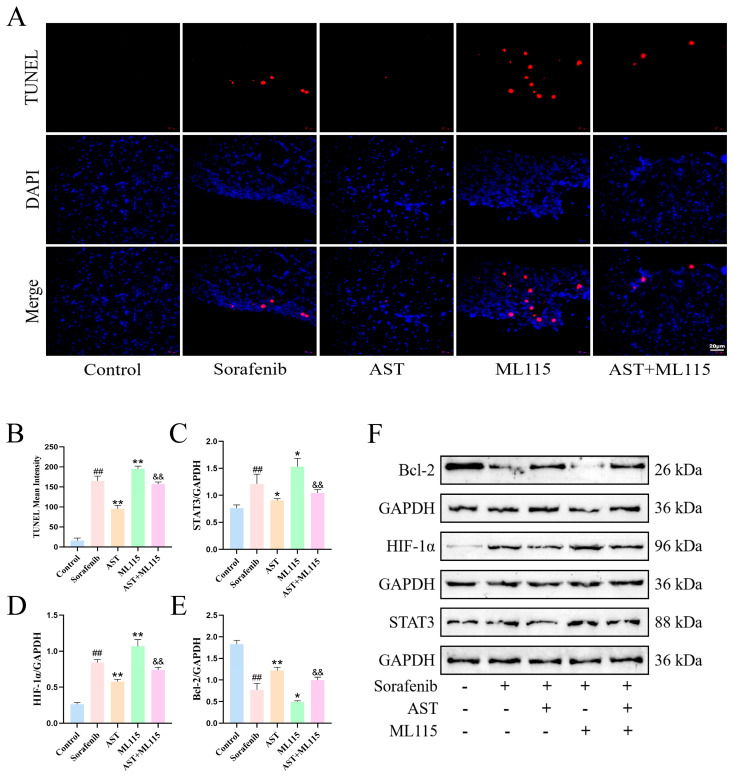
AST attenuated SOR-induced cardiomyocyte injury and oxidative stress. (**A**,**B**) Representative illustrations of treated cardiomyocytes (scale bars = 20 µm) and quantitative analysis of TUNEL mean fluorescence intensity, *n* = 3. (**C**–**F**) Representative Western blot of Bcl-2, HIF-1α, and STAT3, *n* = 3. ## *p* < 0.01 vs. control; * *p* < 0.05 and ** *p* < 0.01 vs. SOR; && *p* < 0.01 vs. ML115.

TUNEL staining was performed to evaluate cardiomyocyte apoptosis in mouse myocardial tissues, and Western blot was used to detect the expression of STAT3/HIF-1α/Bcl-2 pathway-related proteins in myocardial tissues, with the aim of exploring the anti-apoptotic and cardioprotective mechanisms of AST. As shown in Figure 4A,B, compared with the control group, SOR treatment significantly increased the mean fluorescence intensity (control: 16.05 ± 6.2 vs. sorafenib: 165.06 ± 11.9; ## *p* < 0.01), indicating that SOR induced prominent cardiomyocyte apoptosis in mice. AST co-treatment markedly reduced these indicators (AST: 95.6 ± 7.8; ** *p* < 0.01 vs. sorafenib), suggesting that AST effectively inhibits SOR-induced cardiomyocyte apoptosis. Pretreatment with ML115 (a specific STAT3 agonist) (ML115: 195.08 ± 6.88; ** *p* < 0.01 vs. sorafenib) reversed AST’s anti-apoptotic effect, leading to a significant increase in apoptotic cells (AST + ML115: 157.13 ± 5.29; && *p* < 0.01 vs. ML115).

The results of the Western blot showed that compared with the control group, SOR treatment significantly upregulated the protein expression of STAT3 (control: 0.76 ± 0.06 vs. sorafenib: 1.21 ± 0.18; ## *p* < 0.01) and HIF-1α (control: 0.26 ± 0.02 vs. sorafenib: 0.84 ± 0.04; ## *p* < 0.01), while it downregulated Bcl-2 (control: 1.83 ± 0.09 vs. sorafenib: 0.77 ± 0.15; ## *p* < 0.01) in cardiomyocytes (Figure 4C–F). Relative to the SOR group, AST treatment effectively inhibited the SOR-induced upregulation of STAT3 (AST: 0.91 ± 0.03; * *p* < 0.05 vs. sorafenib) and HIF-1α (AST: 0.58 ± 0.03; ** *p* < 0.01 vs. sorafenib) and increased Bcl-2 expression (AST: 1.22 ± 0.08; ** *p* < 0.01 vs. sorafenib). Notably, ML115 treatment yielded results opposite to those of AST: it further promoted the upregulation of STAT3 (ML115: 1.53 ± 0.15; * *p* < 0.05 vs. sorafenib) and HIF-1α (ML115: 1.07 ± 0.09; ** *p* < 0.01 vs. sorafenib) and reduced Bcl-2 expression (ML115: 0.49 ± 0.02; * *p* < 0.05 vs. sorafenib) under SOR treatment, and pretreatment with ML115 abolished AST’s regulatory effects on these proteins (AST + ML115 STAT3: 1.05 ± 0.06; AST + ML115 HIF-1α: 0.74 ± 0.04; AST + ML115 Bcl-2: 1 ± 0.07; && *p* < 0.01 vs. ML115).

### 2.5. AST Alleviated the Damage of HL-1 Induced by SOR

**Figure 5:**
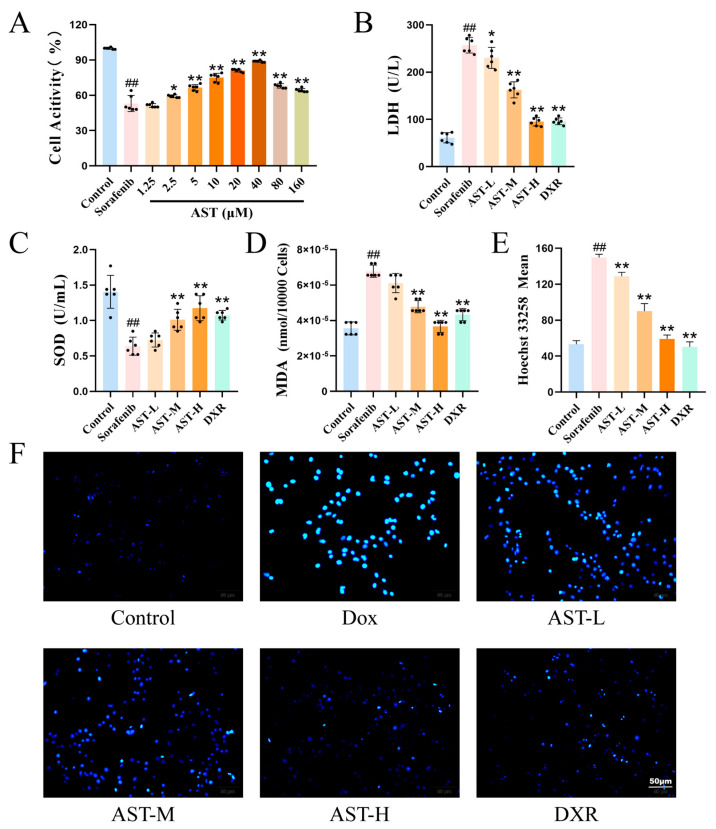
AST protected against SOR−induced cardiomyocyte injury in HL−1 cells. (**A**) Cardiomyocyte viability assessed after treatment with SOR alone or in combination with different concentrations of AST (1.25−160 µM), *n* = 6. (**B**−**D**) Quantification of LDH release, SOD activity, and MDA content, *n* = 6 (AST−L, 10 µM), (AST−M, 20 µM), and (AST−H, 40 µM). (**E**,**F**) Hoechst 33258 staining in cardiomyocytes: mean fluorescence intensity and representative microscopy images. Scale bars = 50 µm, *n* = 6. ## *p* < 0.01 vs. control; * *p* < 0.05, ** *p* < 0.01 vs. SOR group.

To investigate the protective effect of AST on SOR-induced HL-1 cell damage, HL-1 cells were treated with AST at gradient concentrations, and cell viability was detected. As shown in Figure 5A, SOR treatment led to a significant reduction in HL-1 cell viability compared with the control group (control: 99.83 ± 0.75% vs. sorafenib: 53 ± 6.81%; ## *p* < 0.01), while AST treatment at various concentrations improved cell viability to different extents (2.5 µM: 59.17 ± 1.17%; 5 µM: 66.5 ± 2.59%; 10 µM: 75 ± 3.63%; 20 µM: 81 ± 1.26%; 40 µM: 88.83 ± 0.75%; 80 µM: 68 ± 1.9%; 160 µM: 64.33 ± 1.51%; * *p* < 0.05 and ** *p* < 0.01 vs. sorafenib).

Subsequently, low (AST-L, 10 µM), medium (AST-M, 20 µM), and high (AST-H, 40 µM) doses of AST were selected to detect cell injury and oxidative stress indicators. As shown in Figure 5B–D, SOR treatment significantly elevated the levels of LDH (control: 61.29 ± 10.95 U/L vs. sorafenib: 257.1 ± 16.58 U/L; ## *p* < 0.01) and MDA (control: 3.56 × 10^−5^ ± 3.65 × 10^−6^ nmol/10,000 cells vs. sorafenib: 6.78 × 10^−5^ ± 3.44 × 10^−6^ nmol/10,000 cells; ## *p* < 0.01) and decreased the activity of SOD (control: 1.41 ± 0.23 U/mL vs. sorafenib: 0.64 ± 0.13 U/mL; ## *p* < 0.01) compared with the control group. In contrast, AST treatment at different doses reversed these changes: AST reduced LDH (AST-L: 230.32 ± 22.19 U/L; AST-M: 162.58 ± 16.99 U/L; AST-H: 95.16 ± 9.06 U/L; * *p* < 0.05 and ** *p* < 0.01 vs. sorafenib) and MDA levels (AST-M: 4.78 × 10^−5^ ± 3.44 × 10^−6^ nmol/10,000 cells; AST-H: 3.67 × 10^−5^ ± 3.44 × 10^−6^ nmol/10,000 cells; ** *p* < 0.01 vs. sorafenib) and increased SOD activity (AST-M: 1.01 ± 0.15 U/mL; AST-H: 95.16 ± 9.06 U/mL; ** *p* < 0.01 vs. sorafenib) in HL-1 cells, with the effect of AST-H being comparable to that of the positive control drug DXR.

### 2.6. AST Alleviated SOR-Induced HL-1 Cell Apoptosis Through the STAT3/HIF-1α/Bcl-2 Pathway

**Figure 6:**
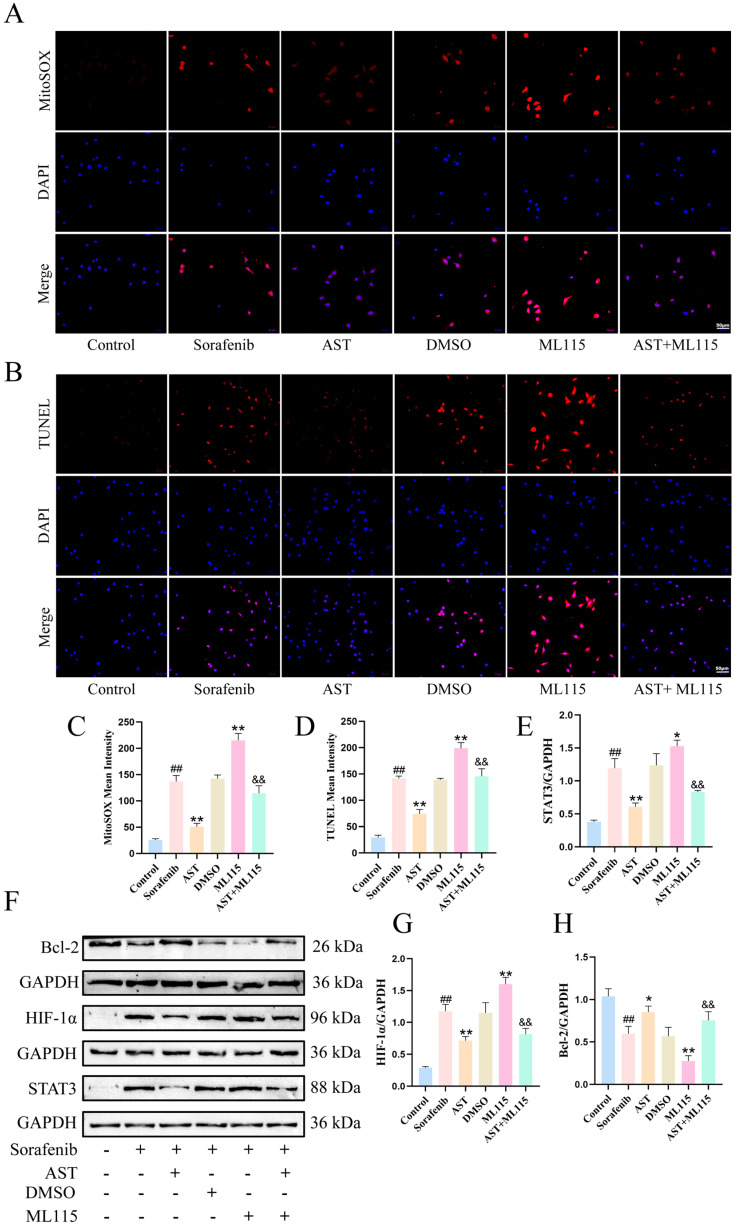
AST alleviates SOR-induced mitochondrial oxidative stress and apoptosis through the STAT3/HIF-1α/Bcl-2 pathway. (**A**,**C**) Representative fluorescence images of MitoSOX staining, *n* = 3. Scale bars = 50 µm. (**B**,**D**) Representative images of TUNEL staining, *n* = 3. Scale bars = 50 µm (AST, 40 µM). (**E**–**H**) Representative Western blot images of STAT3, HIF-1α, and Bcl-2 protein expression, *n* = 3. ## *p* < 0.01 vs. control; * *p* < 0.05, ** *p* < 0.01 vs. SOR; && *p* < 0.01 vs. ML115.

To evaluate the effect of AST on SOR-induced HL-1 cell apoptosis, Hoechst staining, MitoSOX staining, and TUNEL staining were performed. As shown in Figure 5E,F, SOR treatment significantly increased the fluorescence intensity of Hoechst 33258 in HL-1 cells (control: 53.23 ± 4.06 vs. sorafenib: 149.7 ± 3.53; ## *p* < 0.01), reflecting the nuclear condensation and fragmentation characteristic of apoptosis. AST treatment at different doses reduced this fluorescence intensity (AST-L: 128.81 ± 4.45; AST-M: 90.11 ± 8.39 U/L; AST-H: 59.32 ± 4.22; ** *p* < 0.01 vs. sorafenib), with the anti-apoptotic effect of AST-H being most pronounced and similar to that of DXR (DXR: 50.21 ± 5.77; ** *p* < 0.01 vs. sorafenib). Therefore, a dose of 40 µM of AST was adopted in the subsequent experiments. MitoSOX staining and quantitative analysis showed that SOR treatment significantly increased the red fluorescence intensity compared with the control group (control: 25.78 ± 2.2 vs. sorafenib: 136.9 ± 11.31; ## *p* < 0.01), indicating enhanced mitochondrial oxidative stress (Figure 6A, C). AST co-treatment markedly reduced the mitochondrial oxidative stress level (AST: 50.95 ± 6.15; ** *p* < 0.01 vs. sorafenib). Consistent with these findings, the TUNEL assay demonstrated that SOR treatment significantly elevated the mean fluorescence intensity (control: 29.43 ± 4.07 vs. sorafenib: 141.55 ± 4.43; ## *p* < 0.01), while AST treatment reduced the mean fluorescence intensity (AST: 74.04 ± 8.63; ** *p* < 0.01 vs. sorafenib) (Figure 6B,D).

To clarify the molecular mechanism underlying this protection, Western blot analysis was performed (Figure 6E–H). The results revealed that SOR treatment downregulated the expression of Bcl-2 (control: 1.04 ± 0.09 vs. sorafenib: 0.6 ± 0.09; ## *p* < 0.01). In contrast, AST co-treatment decreased the expression of STAT3 (AST: 0.61 ± 0.06 vs. sorafenib: 1.19 ± 0.15; ** *p* < 0.01) and HIF-1α (AST: 0.71 ± 0.07 vs. sorafenib: 1.17 ± 0.11; ** *p* < 0.01), while it also increased the expression of Bcl-2 (AST: 0.85 ± 0.07 vs. sorafenib: 0.6 ± 0.09; * *p* < 0.05). To verify the specific role of STAT3 in this process, cells were pretreated with ML115 (ML115 STAT3: 1.53 ± 0.09; ML115 HIF-1α: 1.6 ± 0.11; ML115 Bcl-2: 0.28 ± 0.06; * *p* < 0.05, ** *p* < 0.01 vs. sorafenib), a STAT3 agonist, prior to AST and SOR co-treatment. This pretreatment reversed AST’s regulatory effects on STAT3 (AST + ML115 STAT3: 0.83 ± 0.03; && *p* < 0.01 vs. ML115), HIF-1α (AST + ML115 HIF-1α: 0.81 ± 0.09; && *p* < 0.01 vs. ML115), and Bcl-2 protein expression (AST + ML115 Bcl-2: 0.75 ± 0.1; && *p* < 0.01 vs. ML115), as well as its anti-apoptotic action.

## 3. Discussion

SOR-induced chronic myocardial injury is a common adverse reaction limiting its clinical application [29], while AST exhibits definite cardioprotective properties in multiple drug-induced cardiac injury contexts [30]. The dosages of SOR and AST adopted in this study were scientifically and rationally determined: the same dose of SOR that we used has been referenced in multiple published studies [31], and continuous intragastric administration for 28 consecutive days was implemented to establish a stable and reproducible murine model of chronic cardiotoxicity. This long-term treatment protocol precisely mimics the sustained clinical exposure of cancer patients undergoing prolonged SOR antitumor therapy, which fully conforms to the slow and progressive characteristics of clinical TKI-related myocardial injury. The dosage of AST that we utilized was validated previously in our preliminary studies on drug-caused myocardial injury [32], confirming its safe and effective cardioprotective concentration. Collectively, the standardized dosing strategy and chronic modeling system utilized in our work are reasonable and reliable; they provided a credible experimental foundation for exploring the protective effect and mechanism of AST and further enhanced the clinical translational value of the present findings.

In this study, network pharmacology was used as a preliminary predictive approach to screen potential targets, followed by further experimental verification. A key methodological innovation of this study was the integration of network pharmacology and human proteome microarray screening. In this assay, biotin-labeled AST was used as a probe to interact with human recombinant proteins on the microarray, and a total of 848 potential binding proteins were preliminarily screened out. Combined with the predictive data from network pharmacology, this dual-strategy analysis strengthened the scientific rigor of the target screening and improved the clinical translational potential of our findings. This comprehensive screening system ultimately identified STAT3 as a novel direct binding target of AST, which was further verified via molecular docking and molecular dynamics simulation. Mechanistically, we elucidated that the core protective effects are modulated through the inhibition of the STAT3/HIF-1α/Bcl-2 pathway.

Subsequently, regarding the pharmacodynamic effects of AST, in vivo, SOR significantly reduced cardiac function indicators, induced myocardial pathological damage and mitochondrial structural destruction, and increased serum levels of cardiac injury and oxidative stress markers (CK-MB, BNP, MDA, LDH) while decreasing SOD activity, all of which were notably reversed by AST treatment. In vitro, SOR markedly decreased the viability of HL-1 cardiomyocytes and triggered cellular oxidative stress injury, whereas AST at different doses effectively improved cell viability and alleviated SOR-induced oxidative damage in HL-1 cells. These results consistently indicate that AST exerts favorable protective effects against SOR-induced cardiotoxicity, reflecting the cardiovascular protective activity of AST in line with existing reports [33,34,35,36].

**Figure 7:**
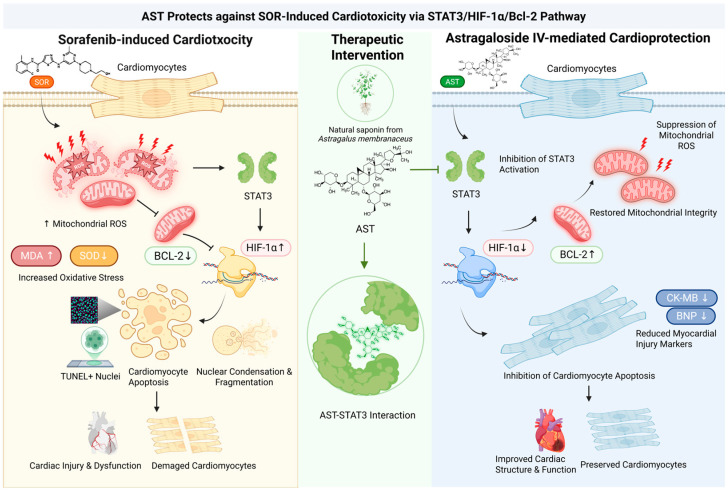
Mechanism diagram of AST ameliorating sorafenib-induced cardiotoxicity.

To explore the mechanism underlying AST-mediated protection against SOR-induced cardiotoxicity, the STAT3 agonist ML115 was used for intervention. In vivo, TUNEL staining and Western blot analysis showed that AST alleviated SOR-induced cardiomyocyte apoptosis and normalized the expression of STAT3/HIF-1α/Bcl-2 pathway-related proteins; however, ML115 treatment further aggravated SOR-triggered cardiomyocyte apoptosis and abnormal protein expression, and pretreatment with ML115 completely reversed the anti-apoptotic and pathway-regulatory effects of AST. In vitro, consistent results were observed in HL-1 cardiomyocytes: AST mitigated SOR-induced mitochondrial oxidative stress and apoptosis by regulating the STAT3/HIF-1α/Bcl-2 pathway, while ML115 blocked the protective effects of AST on SOR-injured HL-1 cells and abrogated its regulatory role in this signaling pathway. Collectively, these results demonstrate that the protective effect of AST against SOR-induced cardiotoxicity is dependent on the inhibition of the STAT3/HIF-1α/Bcl-2 signaling pathway (Figure 7).

Given the key regulatory role of STAT3 identified in our study, extensive attention should be paid to this molecule in the development of cardiotoxicity caused by TKI-class chemotherapeutic agents such as sorafenib, and it may represent a pivotal potential therapeutic target for alleviating TKI-related cardiac injury [37,38,39]. As our findings confirmed that AST can directly bind to STAT3 and block its downstream pathological signaling, it is suggested that AST is a promising candidate drug capable of directly intervening in TKI-induced cardiotoxicity by targeting STAT3.

This study has certain limitations that should be acknowledged when interpreting our findings. Our results demonstrate that AST directly binds to STAT3 and simultaneously reduces the total protein level of STAT3. Although the present study confirmed the direct interaction between AST and STAT3, the precise mechanism by which AST binding leads to decreased STAT3 protein expression remains to be fully elucidated. This reduction may result from enhanced protein degradation, suppressed gene transcription, or inhibited protein translation. Further studies are required to clarify whether direct binding of AST promotes STAT3 degradation or modulates its expression at the transcriptional or translational level. Regarding the potential impact of AST on the antitumor efficacy of sorafenib, this issue represents an important consideration for clinical application. In addition, in the present study, AST protected cardiomyocytes by inhibiting excessive STAT3 activation and reducing cardiomyocyte apoptosis under SOR-induced stress. Given the inherent differences in biological characteristics between tumor cells and cardiomyocytes, the regulatory effect of AST on STAT3 activity in normal cardiomyocytes may not interfere with the antitumor efficacy of sorafenib in malignant tissues. Therefore, whether AST affects tumor growth or sorafenib sensitivity in tumor-bearing models remains unclear and requires further systematic evaluation in future studies.

In conclusion, this study not only confirms the therapeutic potential of AST against SOR-induced cardiotoxicity but also systematically elucidates its novel mechanism of action. Future studies should focus on clinical translational exploration and long-term safety evaluation of combined administration to provide a more theoretical basis for the clinical application of AST combined with SOR.

## 4. Materials and Methods

### 4.1. Chemicals and Materials

Sorafenib (No: HY-10201), astragaloside IV (AST, No: HY-N0431), ML115 (No: HY-111152), and dexrazoxane (DXR, No: HY-B0581) were purchased from MedChemExpress (MCE, Monmouth Junction, NJ, USA). Primary antibodies against STAT3 (#AF6294), HIF1A (#AF1009), Bcl-2 (#AF6139), and GAPDH (#AF7021) were purchased from Affinity Biosciences. The TUNEL Apoptosis Detection Kit (No: C1090) and Hoechst 33258 (No: C1018) were obtained from Beyotime Biotechnology (Shanghai, China). The CCK-8 Kit (No: 40203ES76) was sourced from Yeasen Biotechnology, and the SOD Assay Kit (No: A001-3-2), MDA Assay Kit (No: A003-1-2), and LDH Assay Kit (No: A020-2-2) were acquired from Nanjing Jiancheng Bioengineering Institute (Nanjing, China). The CK-MB Assay Kit (No: JL12422) and BNP Assay Kit (No: JL12884) were obtained from JONLNBIO. HL-1 cardiomyocytes were obtained from BeNa Culture Collection (Henan Engineering Technology Research Center of Industrial Microbial Strains, Zhengzhou, China), with the strain number BNCC288890. Fetal bovine serum (FBS, No: 10099-141) and Dulbecco’s Modified Eagle Medium (DMEM, No: C11995500BT) were procured from Gibco (Gibco Life Sciences, Carlsbad, CA, USA).

### 4.2. Target Prediction and Network Pharmacology Analysis

Potential molecular targets of AST were retrieved from the SwissTargetPrediction database (https://www.swisstargetprediction.ch, accessed on 24 March 2025) and TCMSP (https://old.tcmsp-e.com/tcmsp.php, accessed on 24 March 2025). Disease-related targets for SOR-induced injury were collected from OMIM (https://www.omim.org/, accessed on 24 March 2025), GeneCards (https://www.genecards.org/, accessed on 24 March 2025), and DrugBank (https://go.drugbank.com/, accessed on 24 March 2025), where relevant keywords were utilized. After removing duplicates, the overlapping targets between AST and SOR injury were identified. A protein–protein interaction (PPI) network of the common targets was constructed using the STRING database (https://string-db.org, accessed on 26 March 2025), and the network was further visualized and analyzed using Cytoscape 3.9.1. Kyoto Encyclopedia of Genes and Genomes (KEGG) pathway enrichment analyses and Gene Ontology (GO) for the overlapping targets were conducted with DAVID (https://davidbioinformatics.nih.gov/, accessed on 27 March 2025). The resulting data were visualized using an online bioinformatics platform (https://www.bioinformatics.com.cn/, accessed on 27 March 2025).

### 4.3. Molecular Docking

The crystal conformation of the target polypeptide STAT3 (PDB ID: 6NJS) was retrieved from the Protein Data Bank (https://www.rcsb.org, accessed on 24 April 2025). Water molecules and endogenous ligands were removed from the protein structure using the PyMOL Molecular Graphics System 2.4.0. Subsequently, hydrogen atoms were added to the processed protein using AutoDock Tools 1.5.7 software. The chemical structure of AST was downloaded from the PubChem database (https://pubchem.ncbi.nlm.nih.gov, accessed on 24 April 2025) and converted into a three-dimensional (3D) conformation using ChemBio 3D 14.0. Molecular docking simulations between AST and STAT3 were carried out with AutoDock, and the binding interactions were visualized using PyMOL 2.4.0 and the Proteins plus online tool (https://proteins.plus/, accessed on 24 April 2025).

### 4.4. HuProt™ Human Proteome Microarray

HuProt™ human proteome microarray analysis was performed by Wayen Biotechnologies (Shanghai), Inc. (Shanghai, China) to identify direct binding proteins of AST. Biotin-labeled AST (Bio-AST) was used as the experimental probe, and D-Biotin was used as the negative control. Both samples were dissolved in DMSO and diluted to 10 μM with 1× PBS-T (0.1% Tween-20) before use. The microarray was blocked with 5% BSA in PBS-T at room temperature for 1.5 h and then incubated with Bio-AST or D-Biotin in the dark for 1 h with gentle shaking. After washing with PBS-T and deionized water, the chip was incubated with 0.1% Cy5-Streptavidin solution for 20 min, followed by additional washing steps. The chip was dried via centrifugation at 1000 rpm for 2 min and scanned using a GenePix 4000B microarray scanner at 635 nm. Signal intensities were analyzed using GenePix Pro v6.0 software.

### 4.5. Animals and Treatment

Male C57BL/6 mice (6–8 weeks old, 20–22 g) were obtained from Shanghai SLAC Laboratory Animal Co., Ltd. (Shanghai, China) and housed in a specific pathogen-free (SPF) environment with a 12 h light/dark cycle, 22 ± 2 °C temperature, and 50 ± 5% humidity for 1 week of acclimatization. All animal procedures were approved by the Animal Ethics Committee of Shanghai University of Traditional Chinese Medicine (approval No. PZSHUTCM2505230005). The mice were randomly divided into six groups as follows: control group (*n* = 6), sorafenib (SOR) group (*n* = 12, 50 mg/kg/day), astragaloside IV (AST) group (*n* = 10, 50 mg/kg/day), dexrazoxane (DXR) group (*n* = 10, 40 mg/k/dayg), ML115 (STAT3 agonist) group (*n* = 12, 5 mg/kg/day), and AST + ML115 group (*n* = 10, AST 50 mg/kg/day combined with ML115 5 mg/kg/day). Considering the potential mortality of mice induced by chronic toxic injury, model-related groups were arranged with an appropriate larger sample size, while the animal number in other groups was reasonably arranged to minimize unnecessary animal usage and comply with the 3R principle. Gavage administration was initiated at 13:00 daily. The mice were treated with AST one hour prior to the SOR intervention, and the whole treatment lasted for 28 consecutive days. Echocardiographic measurements were performed at the experimental endpoint (24 h after the last administration) to assess cardiac function, after which the mice were euthanized via CO_2_ inhalation. Following this, blood samples were collected from the abdominal aorta for serum biochemical analysis, and heart tissues were rapidly harvested, rinsed with ice-cold PBS, and then divided into two portions: one fixed in 4% paraformaldehyde for histopathological and TUNEL staining and the other stored at −80 °C for Western blot analysis and oxidative stress marker detection. The treatment dosages of SOR and AST were selected according to those used in previous studies [31,32].

### 4.6. Histopathological and Ultrastructural Analysis

Myocardial tissues were fixed with 4% formaldehyde solution, followed by paraffin embedding and sectioning into 4 μm thick slices for HE and Masson trichrome staining. Collagen content was quantified using Image J 1.53k. For TEM, tissues fixed in 2.5% glutaraldehyde were embedded in epoxy resin, sectioned (60 nm), stained with uranyl acetate–lead citrate, and observed under Hitachi H-7650 TEM.

### 4.7. Cell Culture and Treatment

HL-1 cardiomyocytes were cultured in DMEM medium supplemented with 10% fetal bovine serum and 1% penicillin–streptomycin and maintained in a humidified incubator at 37 °C with 5% CO_2_. For dose-dependent protection assays, cells were pretreated with AST at AST-L (10 μM), AST-M (20 μM), and AST-H (40 μM) doses for 2 h, followed by co-treatment with sorafenib (SOR, 10 μM) for 24 h.

### 4.8. Cell Viability and Biochemical Assays

Cell viability was detected using the CCK-8 Kit (450 nm absorbance). CK-MB, BNP, and LDH levels in the supernatant and SOD activity and MDA content in the cell lysates were measured utilizing commercial kits in accordance with the manufacturer’s instructions.

### 4.9. Apoptosis Assay

TUNEL staining was performed on tissue sections and cells (proteinase K digestion, 37 °C incubation for 60 min, DAPI counterstaining). Hoechst 33258 staining (5 μg/mL, 15 min) was used to observe nuclear morphology.

### 4.10. Western Blot Analysis

Total protein was extracted with RIPA lysis buffer (with protease/phosphatase inhibitors). Equal protein (30 μg) was separated by 10% SDS-PAGE, transferred to PVDF membranes, blocked with 5% non-fat milk, and incubated with primary antibodies (1:1000) at 4 °C overnight and secondary antibodies (1:5000) at room temperature for 1 h. Bands were visualized using ECL and quantified via Image J 1.53k.

### 4.11. Statistical Analysis

All experimental data are presented as mean ± standard deviation (SD). A one-way analysis of variance (ANOVA) with Tukey’s post hoc test was subsequently used for comparing means among multiple groups, while comparisons between the two groups were performed using an independent-sample t-test. In all statistical analyses, a *p*-value < 0.05 was considered to be statistically significant.

## 5. Conclusions

In summary, AST mitigates SOR-induced cardiotoxicity by attenuating cardiomyocyte apoptosis and oxidative stress. The underlying molecular mechanism may include the inhibition of the STAT3/HIF-1α/Bcl-2 pathway.

## Data Availability

The data that support the findings of this study are available upon reasonable request from the corresponding author.

## Abbreviations

The following abbreviations are used in this manuscript:

AST	Astragaloside IV
SOR	Sorafenib
STAT3	Signal Transducer and Activator of Transcription 3
HIF-1α	Hypoxia-Inducible Factor-1α
Bcl-2	B-cell Lymphoma-2
TKIs	Tyrosine Kinase Inhibitors
TUNEL	Terminal Deoxynucleotidyl Transferase dUTP Nick-End Labeling
SOD	Superoxide Dismutase
MDA	Malondialdehyde
CK-MB	Creatine Kinase MB Isoenzyme
BNP	Brain Natriuretic Peptide
PI3K	Phosphatidylinositol 3-kinase
LDH	Lactate dehydrogenase
PPI	Protein–Protein Interaction
GO	Gene Ontology
KEGG	Kyoto Encyclopedia of Genes and Genomes
RMSD	Root Mean Square Deviation
LVEF	Left Ventricular Ejection Fraction
LVFS	Left Ventricular Fractional Shortening
DXR	Dexrazoxane
